# The clinical significance of peripheral blood cell ratios in patients with intracranial aneurysm

**DOI:** 10.3389/fneur.2022.1080244

**Published:** 2022-12-20

**Authors:** Hyun Kyung Kim, Kee Ook Lee, Seung-Hun Oh, Kyung-Yul Lee, Seung-Wook Choo, Ok Joon Kim, Tae Gon Kim, Sang-Heum Kim, Sang-Jun Na, Ji Hoe Heo

**Affiliations:** ^1^Department of Neurology, CHA Bundang Medical Center, School of Medicine, CHA University, Seongnam-si, South Korea; ^2^Departments of Neurology, Yonsei University College of Medicine, Seoul, South Korea; ^3^Department of Biomedical Laboratory Science, College of Natural Science, Daejeon University, Daejeon, South Korea; ^4^Department of Neurosurgery, CHA Bundang Medical Center, School of Medicine, CHA University, Seongnam-si, South Korea; ^5^Department of Radiology, CHA Bundang Medical Center, School of Medicine, CHA University, Seongnam-si, South Korea; ^6^Department of Neurology, Konyang University College of Medicine, Daejeon, South Korea

**Keywords:** blood cell ratio, rupture risk, intracranial aneurysm, inflammation, biomarkers

## Abstract

**Background and objective:**

Inflammation is an important factor in the development of aneurysm, and has been identified as a key characteristic predictive of rupture of intracranial aneurysm (IA). However, the role of inflammatory peripheral blood cell ratios in patients with IA has not been well delineated.

**Methods:**

A total of 1,209 patients, including 1,001 with unruptured IA and 208 with ruptured IA, were enrolled in this study. Neutrophil-to-lymphocyte ratio (NLR), platelet-to-lymphocyte ratio (PLR), platelet-to-neutrophil ratio (PNR), and platelet-to-white-blood-cell ratio (PWR) were compared between ruptured and unruptured IA.

**Results:**

Compared with the ruptured IA group, the unruptured IA group had higher PNR {median, 65.96 [interquartile range (IQR) 48.95–85.05] vs. 37.78 (IQR, 23.17–54.05); p < 0.001} and PWR [median, 36.89 (IQR 29.38–44.56) vs. 22.39 (IQR, 16.72–29.29); p < 0.001]. In multivariate analysis, PNR and PWR were independently associated with ruptured IA (*p* = 0.001 and *p* < 0.001, respectively). Unruptured IA subgroup analyses according to the PHASES scores showed that a higher PHASES score was associated with significantly higher NLR and erythrocyte sedimentation rate (*p* < 0.001 and *p* = 0.025) and lower PNR and PWR (*p* < 0.001 and *p* = 0.007).

**Conclusions:**

We demonstrated that lower PNR and PWR levels are associated with ruptured IA and a higher PHASES score. Unlike many other inflammatory markers and bioassays, peripheral blood cell ratios are inexpensive and readily available biomarkers that may be useful for risk stratification in patients with cerebral aneurysm. However, a long-term prospective study is needed to clarify this matter.

## Introduction

The prevalence of unruptured intracranial aneurysm (IA) in the general population is ~3–5% (1, 2). The detection of unruptured IA is increasing because of the wider availability of imaging techniques (2, 3). Complications associated with IA include a mass effect on adjacent structures (cranial nerves, brain stem, etc.) (4); rupture is the most severe and frequent aneurysm complication. The estimated incidence of this latter complication is ~1–2% per aneurysm per year (5). However, such ruptures can cause subarachnoid hemorrhage (SAH), which has an initial mortality rate of 10–15%. Of patients who are able to reach medical care, 25% die within the first 2 weeks, while 20–30% of the survivors are severely and permanently disabled (6). In contrast, IA that is repaired before rupture by microsurgical clipping or endovascular coiling has a reported mortality rate as low as 0.6% and morbidity of <5% (7, 8). There is thus a need to discover reliable additional predictive markers for improved risk assessment and screening of individuals for the development or rupture of an IA.

Inflammation is an important factor in the development of aneurysm, and has been identified as an important characteristic predictive of rupture of IA (9, 10). Inflammation is also an important factor in aneurysm remodeling, which can in turn cause aneurysm growth and rupture (11). Infiltration of neutrophils has also been found to be greater in ruptured vs. unruptured IA (12, 13).

Total white blood cell (WBC) count and the counts of subtypes, such as neutrophils, lymphocytes, and the neutrophil-to-lymphocyte ratio (NLR), can be used as indicators of systemic inflammation (14). NLR, which was first reported in 1967, is a marker of subclinical inflammation used in many studies, which is both simple and inexpensive to use. NLR has recently emerged as a prognostic marker in patients with cancer and coronary artery disease (14, 15). The platelet-to-lymphocyte ratio (PLR) has also been proposed as a biomarker for inflammatory diseases and shown to be related to many medical disorders (16, 17). However, little is known about the roles of NLR and PLR in patients with IA. In addition, many studies have clearly verified the relationship between other blood cell ratios and IA. Therefore, the purpose of this study was to determine the clinical significance of NLR, PLR, platelet-to-neutrophil ratio (PNR), and platelet-to-WBC ratio (PWR) in patients with IA.

## Materials and methods

### Study population

The study group consisted of patients with IA who visited the Department of Neurology and/or Neurosurgery at CHA Bundang Medical Center, Seongnam, Rebublic of Korea, from July 2015 to December 2020. A retrospective analysis was designed for subjects who arrived for medical attention because they had underlying cardiovascular risk factors or a family history of stroke, intracranial hemorrhage, or SAH. Medical records, laboratory results, and radiological findings were reviewed in all subjects. Only participants whose records contained adequate information on demographic, laboratory, and radiological data were included. We did not include patients with recent infection history, clinical fever higher than 37.2°C, or any other neurologic disorders. Of 1,502 study patients extracted from our database during the study period, we excluded 293 for the following reasons: (1) inadequate medical information (*n* = 32); (2) no laboratory tests performed (*n* = 148); (3) no data on brain magnetic resonance imaging (MRI) or magnetic resonance angiography (MRA) (*n* = 13); (4) previous history of neurological disease (*n* = 57); or (5) abnormal clinical and neurological findings at the time of examination (*n* = 43). A total of 1,209 participants were included in the subsequent analysis.

This study was conducted in accordance with the Declaration of Helsinki and approved by the Institutional Ethical Committee of CHA Bundang Medical Center (IRB approval no. CHAMC 2022-04-012).

### Risk factor assessment

We reviewed patients' medical records to gather information on their medical history and laboratory data related to IA. We assessed the previous medical history of IA, cardiac diseases (e.g., atrial fibrillation, myocardial infarction, and valvular heart disease), hypertension (HTN), diabetes mellitus (DM), and smoking in all subjects. HTN was diagnosed when a patient had systolic blood pressure ≥140 mmHg, diastolic blood pressure ≥90 mmHg, or was receiving antihypertensive treatment. DM was diagnosed when a patient had a fasting plasma glucose level ≥7.0 mmol/L, hemoglobin A1C (HbA1c) ≥6.5%, or was receiving oral hypoglycemic agents or insulin (18). Smoking was defined in those who smoked at the time of the examination. Hypercholesterolemia was defined as fasting serum total cholesterol ≥220 mg/dL or a history of statin medication. Cardiovascular diseases included coronary heart disease, heart failure, and peripheral arterial disease. Data indicating coronary arterial occlusive disease (CAOD) were defined as reflecting a history of CAOD and percutaneous coronary interventions or coronary artery bypass grafting. Chronic kidney disease (CKD) was defined as an estimated glomerular filtration rate (eGFR) <60 mL/min/1.73 m^2^. Exclusion criteria were severe hepatic dysfunction (alanine aminotransferase or aspartate aminotransferase >2 times higher than the upper limit of normal value), renal dysfunction (serum creatinine >1,41.4 μmol/L), recent infection history, malignancy, previous history of autoimmune disease, or any other neurological disorder. Patients who had taken medicine that can potentially interfere with the measurement of leukocyte counts, such as anti-inflammatory agents, were also excluded from this study.

### Data collection and laboratory examinations

All blood samples were obtained before initiating treatment. Plasma sample data were collected for all study participants and included only tests performed within 1 day of the radiological examination. Serum specimens were centrifuged, frozen within 2 h of collection, and stored at −80°C until they were analyzed. Serum total cholesterol, high-density-lipoprotein cholesterol, low-density-lipoprotein cholesterol, and triglyceride levels were measured by conventional methods. Total and differential leukocyte counts were measured using an automated hematology analyzer. Absolute cell counts were used in the analyses. Laboratory parameters such as fasting blood glucose, total cholesterol, triglycerides, WBC count, neutrophil count, lymphocyte count, and eGFR were tested in the biochemistry department of the hospital. eGFR was calculated using the abbreviated Modification of Diet in Renal Disease Study Equation [186 × serum creatinine−1.154 × age−0.203 × 0.742 (if female)] (19). Estimated neutrophil and lymphocyte counts were calculated from the differential count of complete blood cell counts, presented as the percentage of the whole WBC count. All tests were performed in one laboratory by a single operator.

### Radiological evaluation

Brain MRI and MRA were performed using four 1.5-T MR and 3-T MR systems (Signa HDX 3.0T, GE Healthcare, Piscataway, NJ, USA; Signa Architect 3.0T, GE Healthcare, Waukesha, WI, USA; Signa HDX 1.5T, GE Healthcare, Waukesha, WI, USA; Ingenia Elition X 3T, Philips Health Systems, Best, NL). Computed tomographic angiography (CTA) was performed using four systems (Resolution CT, GE Healthcare, Waukesha, WI, USA; LightSpeed VCT, GE Healthcare, Waukesha, WI, USA; IQon Spectral CT, Philips Health Systems, Cambridge, MA, USA; Optima 660, GE Healthcare, Chicago, IL, USA). Digital subtraction angiography (DSA) was performed using only one system (Axiom Artis dBA, SIEMENS, Forchheim, DE). Image interpretation was performed by one neurologist, one neurosurgeon, and one radiologist, who were blinded to clinical and laboratory data. The patients with unruptured IA were found incidentally through three-dimensional CTA, MRA, and DSA performed for reasons other than suspicion of an index aneurysm and did not present any IA-related clinical symptoms or signs. The ruptured IA group was selected among aneurysmal hemorrhage patients with a diagnosis of IA using CTA, MRA, and DSA. We only included individuals who underwent CTA, MRA, and/or DSA. The severity of rupture risk of unruptured IA was assessed using the PHASES scoring system (20, 21). Unruptured IA was classified into low- (PHASES score <5), medium- (PHASES score 5–9), and high-rupture-risk (PHASES score>9) subgroups.

### Statistical analysis

To evaluate the factors associated with the blood cell ratios, participants were categorized into ruptured and unruptured IA groups. Baseline characteristics were compared between these groups. Continuous variables are reported as mean ± standard deviation, whereas categorical variables are reported as frequency and percentage. Statistical analysis was performed using SPSS (version 23.0; SPSS Inc., IL, USA). Student's *t*-test was used to compare continuous variables between the groups. Chi-squared and Fisher's exact tests were used to compare categorical variables. In-group analyses were performed with Kruskal-Wallis one-sided analysis of variance and Mann-Whitney *U* test for multiple groups when groups were non-parametrically distributed and sample sizes were unequal. Logistic regression was used to determine the association between blood cell ratios and aneurysm rupture. Adjustments were performed for the following established cardiovascular and IA risk factors: age, sex, HTN, DM, hypercholesterolemia, current smoking status, alcohol, cardiovascular disease, family history of SAH, eGFR, multiplicity, daughter sac, aneurysm size, and aneurysm location. Odds ratios (ORs) and 95% confidence intervals (CIs) were calculated. Receiver operating characteristic (ROC) curve analysis was conducted to determine the area under the curve (AUC), sensitivity, specificity, and cut-off point for the NLR, PLR, PNR, and PWR that optimally predicted IA rupture risk. A two-sided *p* < 0.05 was considered statistically significant.

## Results

The clinical and biochemical baseline characteristics of the study participants are summarized in Table 1. The clinical characteristics of the 1,209 participants included in this study were analyzed according to the presence or absence of IA rupture. There were significant differences between the ruptured and unrputured IA groups with regard to demographic data and vascular risk factor frequency. Compared with the unruptured IA group, ruptured IA group patients were younger and more likely to be male. The levels of eGFR and glucose were significantly higher in the ruptured IA group (both *p* < 0.001). Meanwhile, the rates of HTN, DM, cardiovascular disease, hypercholesterolemia, and CKD were significantly higher in the unruptured IA group (*p* < 0.001, *p* = 0.002, *p* = 0.035, *p* < 0.001, and *p* = 0.019, respectively). In addition, the rates of current smoking, alcohol consumption, and a family history of SAH were significantly higher in the ruptured IA group (*p* < 0.001, *p* < 0.001, and p = 0.007, respectively). In the ruptured IA group, WBC, red blood cell (RBC), hemoglobin (Hb), platelet, neutrophil, and lymphocyte counts were significantly higher (*p* < 0.001, *p* = 0.038, *p* = 0.049, *p* = 0.020, *p* < 0.001, and *p* < 0.001, respectively). The PNR and PWR were significantly higher and the NLR was lower in the unruptured IA group than in the ruptured IA group (Table 1).

**Table 1:** The distribution of demographic and clinical characteristics among unruptured and ruptured IA.

	**Unruptured IA** **(*n* = 1,001)**	**Ruptured IA** **(*n* = 208)**	***P*-value**
Age, years	63.5 ± 12.9	55.3 ± 14.2	<0.001
Sex, female	722 (72.1)	126 (60.6)	0.001
Height, m	1.58 ± 0.09	1.63 ± 0.09	<0.001
Weight, kg	61.2 ± 11.0	65.1 ± 12.1	<0.001
Body mass index, kg/m^2^	24.4 ± 3.6	24.5 ± 4.2	0.532
Hypertension	542 (54.1)	82 (39.4)	<0.001
Diabetes mellitus	189 (18.9)	21 (10.1)	0.002
Smoking, current	174 (17.4)	74 (35.6)	<0.001
Alcohol, current	229 (22.9)	98 (47.1)	<0.001
Cardiovascular disease^*^	101 (10.1)	11 (5.3)	0.035
Hypercholesterolemia	248 (24.8)	19 (9.1)	<0.001
Atrial fibrillation	25 (2.5)	5 (2.4)	1.000
CKD	105 (10.5)	11 (5.3)	0.019
Family history of SAH	13 (1.3)	9 (4.3)	0.007
Total cholesterol, mmol/L	4.53 (3.83–5.26)	4.61 (3.97–5.28)	0.217
Triglyceride, mmol/L	1.34 (0.96–1.94)	1.42 (1.02–2.15)	0.094
HDL-cholesterol, mmol/L	1.34 (1.10–1.64)	1.30 (1.08–1.65)	0.612
LDL-cholesterol, mmol/L	2.49 (1.84–3.21)	2.54 (1.84–3.13)	0.766
Glucose, mmol/L	6.16 (5.49–7.33)	8.38 (7.06–10.43)	<0.001
HbA1c, %	6.89 ± 5.67	6.14 ± 0.87	0.382
eGFR, mL/min/1.73 m^2^	92.26 ± 27.42	101.95 ± 26.94	<0.001
multiplicity	165 (16.5)	49 (23.6)	0.021
Daughter sac	13 (1.3)	17 (8.2)	<0.001
**Size of aneurysm, mm**			<0.001
0–1	113 (11.3)	3 (1.4)	
2–4	677 (67.6)	78 (37.5)	
5–6	130 (13.0)	54 (26.0)	
7–9	54 (5.4)	43 (20.7)	
10–19	25 (2.5)	29 (13.9)	
≥20	2 (0.2)	1 (0.5)	
**Location of aneurysm**			<0.001
MCA	236 (23.6)	53 (25.5)	
ACA	161 (16.1)	76 (36.5)	
ICA	439 (43.9)	18 (8.7)	
ICA-Pcom	105 (10.5)	43 (20.7)	
BA	34 (3.4)	12 (5.8)	
VA	6 (0.6)	0 (0.0)	
Other	20 (2.0)	6 (2.9)	
WBC, 10^9^/L	6.32 (5.26–7.65)	10.87 (8.26–13.75)	<0.001
RBC, 10^12^/L	4.27 (3.95–4.59)	4.35 (4.01–4.68)	0.038
Hb, g/L	131.38 ± 15.80	133.81 ± 17.86	0.049
Platelet, 10^9^/L	235.19 ± 59.50	245.94 ± 63.34	0.020
Neutrophil, 10^9^/L	3.89 ± 1.72	7.74 ± 4.21	<0.001
Lymphocyte, 10^9^/L	2.01 (1.57–2.50)	2.23 (1.48–3.87)	<0.001
PNR	65.96 (48.95–85.05)	37.78 (23.17–54.05)	<0.001
NLR	1.71 (1.27–2.43)	2.58 (1.25–6.59)	<0.001
PLR	115.31 (89.85–147.53)	100.60 (61.57–163.52)	0.002
PWR	36.89 (29.38–44.56)	22.39 (16.72–29.29)	<0.001
ESR, mm/h	17.67 ± 15.88	16.06 ± 14.44	0.196
CRP, mg/L	3.78 ± 11.43	3.86 ± 7.70	0.933

Data are expressed as mean ± SD or median (IQR), number (%).

IA, intracranial aneurysm; CKD, chronic kidney disease; SAH, subarachnoid hemorrhage; HDL, high-density lipoprotein; LDL, low-density lipoprotein; HbA1c, hemoglobin A1c; eGFR, estimated glomerular filtration rate; MCA, middle cerebral artery; ACA, anterior cerebral artery; ICA, internal carotid artery; Pcom, posterior communicating artery; BA, basilar artery; WBC, white blood cell; RBC, red blood cell; Hb, hemoglobin; PNR, platelet-to-neutrophil ratio; NLR, neutrophil-to-lymphocyte ratio; PLR, platelet-to-lymphocyte ratio; PWR, platelet-to-white blood cell ratio; ESR, erythrocyte sedimentation rate; CRP, C-reactive protein.

* Cardiovascular disease includes coronary heart disease, heart failure, or peripheral arterial disease.

From radiological examinations, the rates of multiplicity and daughter sac were significantly higher in the ruptured IA group (*p* = 0.021 and *p* < 0.001, respectively). In addition, the aneurysms tended to be larger in the ruptured IA group. Meanwhile, middle cerebral artery, anterior cerebral artery, posterior communicating artery, and basilar artery aneurysms were more frequently observed in the ruptured IA group.

**Figure 1:**
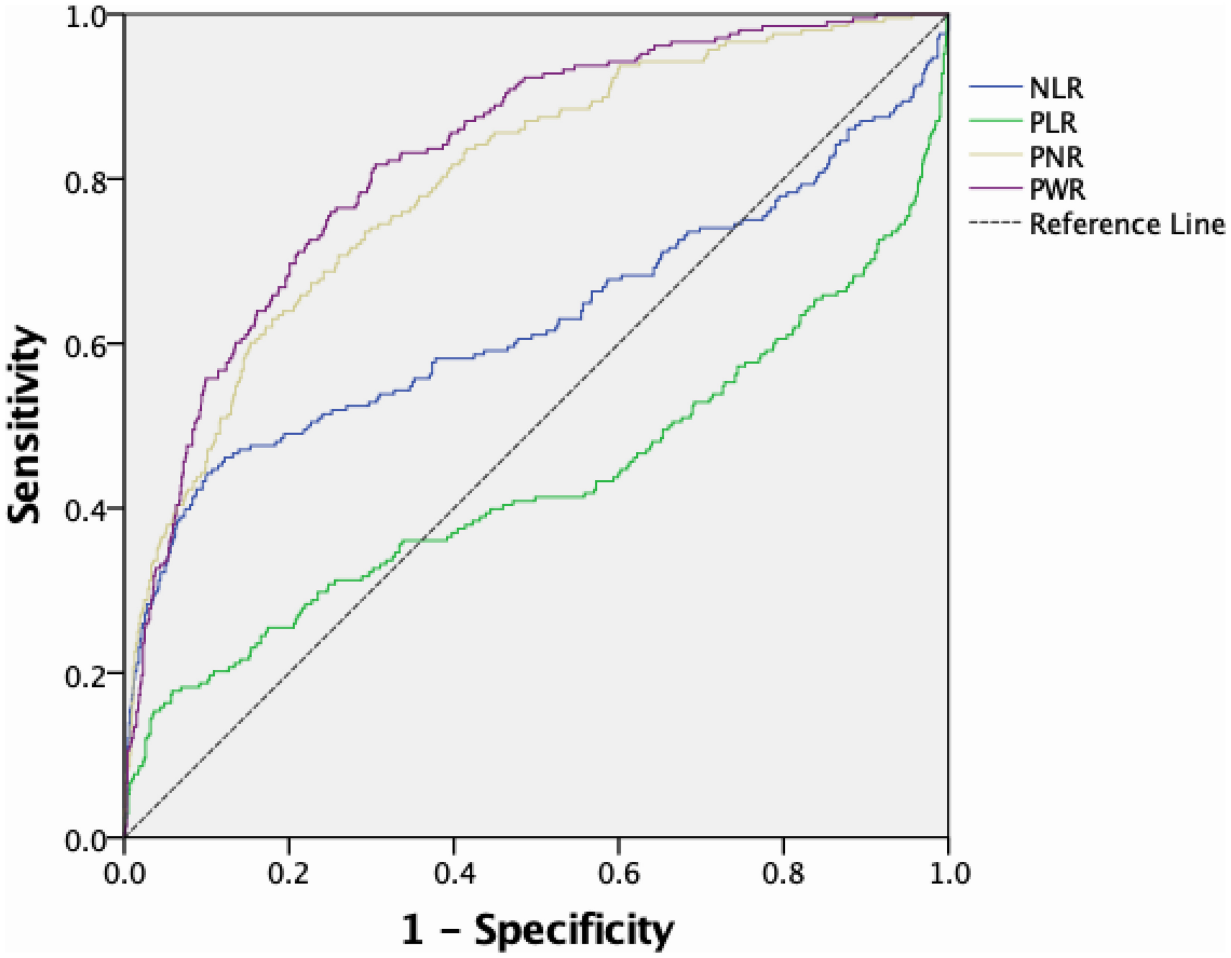
ROC curve and corresponding AUC of peripheral blood cell ratios predicting IA rupture. ROC, Receiver operating characteristic; AUC, area under the curve; IA, intracranial aneurysm.

The ROC curves of the predictive value of each blood cell ratio for IA rupture are shown in Figure 1. PNR could discriminate ruptured IA (AUC, 0.799, *p* < 0.001; cut-off value, 43.95, 63.0% sensitivity, 82.0% specificity) from unruptured IA and PWR could also distinguish ruptured IA (AUC, 0.827, *P* < 0.001; cut-off value, 31.10, 81.7% sensitivity, 69.5% specificity) from unruptured IA. The AUC of NLR was 0.626, the optimal cut-off value was 2.28, the sensitivity was 52.4%, and the specificity was 71.0%. Meanwhile, the AUC of PLR was 0.432, the optimal cut-off value was 107.56, the sensitivity was 43.3%, and the specificity was 42.3%.

We next conducted logistic regression analyses to determine whether blood cell ratios are independently associated with IA rupture. Logistic regression analysis was performed with each of the blood cell ratios, which were included as binary variables dichotomized according to the optimal cut-off value. In our multivariate analyses, the risk of IA rupture was negatively correlated with PNR and PWR (Table 2). PNR and PWR remained significant after adjusting for confounders including cardiovascular and IA risk factors.

**Table 2:** Multivariate adjusted odds ratios for the association between blood cell ratios and cerebral aneurysm rupture.

	Model 1^*^	***P***	**Model 2^†^**	***P***	**Model 3^‡^**	***P***	**Model 4^§^**	***P***
	**OR (95% CIs)**		**OR (95% CIs)**		**OR (95% CIs)**		**OR (95% CIs)**	
**PNR**								
<43.95	Ref		Ref		Ref		Ref	
≥43.95	0.439 (0.272–0.709)	0.001	0.440 (0.264–0.734)	0.002	0.426 (0.250–0.727)	0.002	0.356 (0.190–0.668)	0.001
**NLR**								
<2.28	Ref		Ref		Ref		Ref	
≥2.28	1.276 (0.786–2.070)	0.324	1.334 (0.803–2.216)	0.266	1.373 (0.813–2.319)	0.236	1.333 (0.731–2.428)	0.348
**PLR**								
<107.56	Ref		Ref		Ref		Ref	
≥107.56	0.847 (0.544–1.319)	0.464	0.748 (0.471–1.188)	0.219	0.717 (0.445–1.156)	0.172	0.627 (0.370–1.063)	0.083
**PWR**								
<31.10	Ref		Ref		Ref		Ref	
≥31.10	0.190 (0.113–0.319)	<0.001	0.181 (0.105–0.311)	<0.001	0.182 (0.104–0.319)	<0.001	0.250 (0.135–0.464)	<0.001

* Model 1: no adjustment.

† Model 2: adjusted for age and sex.

‡ Model 3: adjusted for age, sex, hypertension, diabetes mellitus, hypercholesterolemia, current smoking status, alcohol, cardiovascular disease, family history of subarachnoid hemorrhage, and estimated glomerular filtration rate.

§ Model 4: adjusted for age, sex, hypertension, diabetes mellitus, hypercholesterolemia, current smoking status, alcohol, cardiovascular disease, family history of subarachnoid hemorrhage, estimated glomerular filtration rate, multiplicity, daughter sac, aneurysm size, and aneurysm location.

OR, odds ratio; CIs, confidence intervals; PNR, platelet-to-neutrophil ratio; NLR, neutrophil-to-lymphocyte ratio; PLR, platelet-to-lymphocyte ratio; PWR, platelet-to-white blood cell ratio; Ref, reference group.

**Figure 2:**
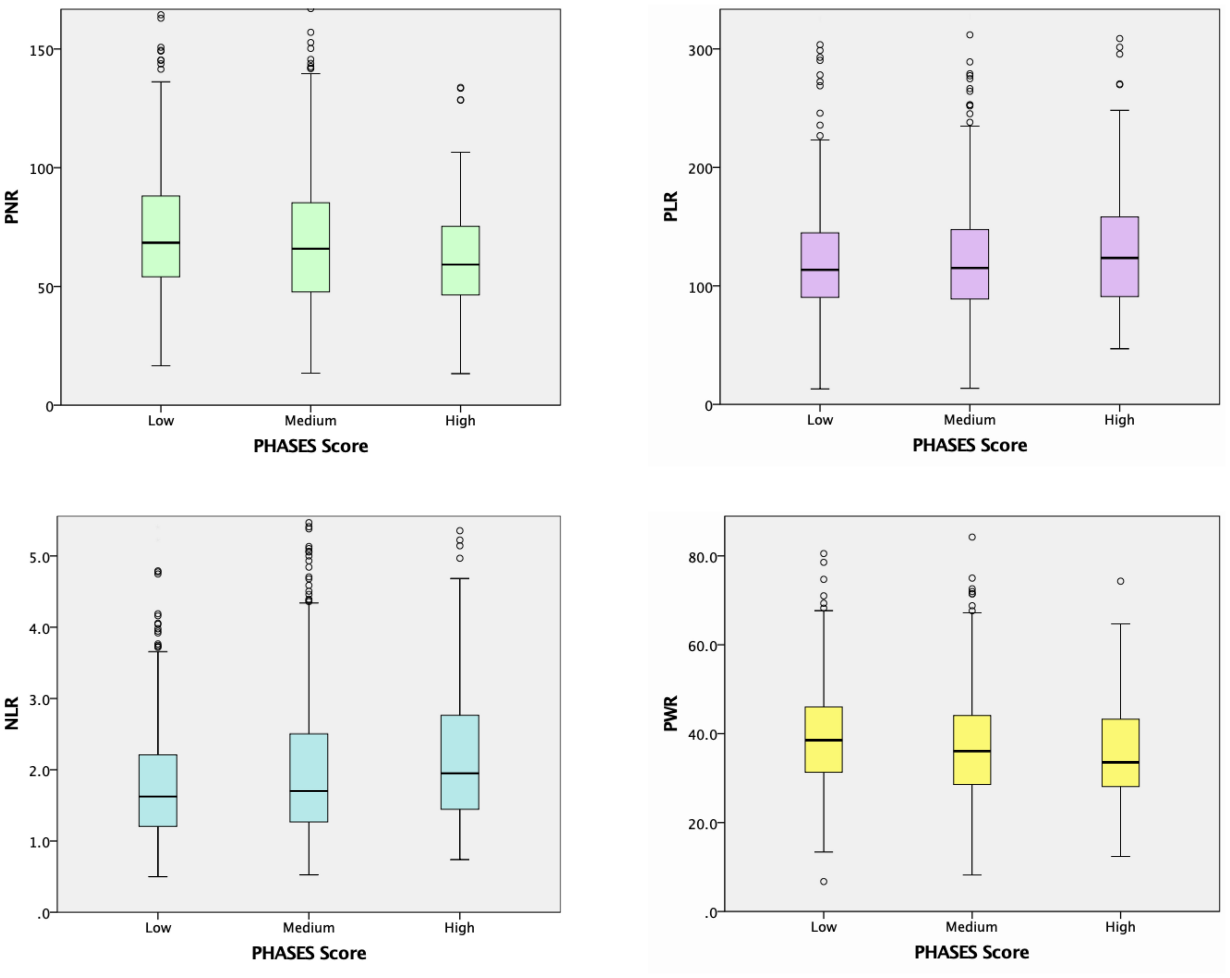
Boxplots of the peripheral blood cell ratios according to PHASES scale score.

Unruptured IA subgroup analyses according to the PHASES score were also performed, as summarized in Table 3. When the baseline demographic and clinical characteristics of the participants were examined, there were significant differences among the three PHASES score groups in terms of age, and rates of HTN, DM, cardiovascular disease, CKD, and alcohol consumption (Table 3). Unruptured IA patients in the group with a higher PHASES score had lower levels of total cholesterol, HDL cholesterol, LDL cholesterol, and eGFR and a higher level of HbA1c. RBC, Hb, and lymphocyte counts were inversely associated with PHASES score, while neutrophil count was positively correlated with it. Subjects with a higher PHASES score showed significantly higher levels of NLR and erythrocyte sedimentation rate (ESR) (*p* = 0.006 and *p* = 0.025) and lower levels of PNR and PWR (*p* = 0.001 and *p* = 0.020) (Table 3; Figure 2).

**Table 3:** Participant characteristics according to the PHASES score.

	**PHASES score**			***P*-value**
	**Low (<5)**	**Medium (5-9)**	**High (9 <)**	
	***n*** = **326**	***n*** = **514**	***n*** = **161**	
Age, years	56.77 ± 12.08	64.89 ± 12.02	72.76 ± 9.71	<0.001
Sex, female	231 (70.9)	369 (71.8)	122 (75.8)	0.508
Systolic blood pressures, mmHg	127.05 ± 15.11	130.35 ± 16.43	130.06 ± 14.70	0.010
Diastolic blood pressures, mmHg	76.24 ± 10.51	76.95 ± 10.54	74.71 ± 10.30	0.062
Height, m	1.60 ± 0.79	1.58 ± 0.85	1.57 ± 0.90	<0.001
Weight, kg	61.29 ± 11.24	61.35 ± 10.67	60.85 ± 11.86	<0.881
Body mass index, kg/m^2^	23.91 ± 3.49	24.55 ± 3.51	24.70 ± 4.07	0.019
Hypertension	97 (29.8)	303 (58.9)	142 (88.2)	<0.001
Diabetes mellitus	35 (10.7)	107 (20.8)	47 (29.2)	<0.001
Smoking, current	62 (19.0)	89 (17.3)	23 (14.3)	0.431
Alcohol, current	90 (27.6)	120 (23.4)	19 (11.8)	<0.001
Cardiovascular disease^*^	21 (6.5)	46 (8.9)	34 (21.1)	<0.001
Hypercholesterolemia	70 (21.5)	16 (26.5)	42 (26.1)	0.252
Atrial fibrillation	5 (1.5)	15 (2.9)	5 (3.1)	0.400
CKD	12 (3.7)	61 (11.9)	32 (19.9)	<0.001
Family history of SAH	5 (1.5)	6 (1.2)	2 (1.2)	0.899
Total cholesterol, mmol/L	4.71 (4.07–5.41)	4.45 (3.76–5.18)	4.20 (3.55–4.91)	<0.001
Triglyceride, mmol/L	1.31 (0.92–1.88)	1.36 (0.99–1.98)	1.37 (0.97–1.84)	0.362
HDL-cholesterol, mmol/L	1.41 (1.18–1.64)	1.31 (1.07–1.64)	1.30 (1.06–1.64)	0.015
LDL-cholesterol, mmol/L	2.59 (2.09–3.34)	2.43 (1.79–3.14)	2.31 (1.61–3.10)	0.007
Glucose, mmol/L	5.99 (5.44–6.83)	6.35 (5.59–7.49)	6.30 (5.55–8.13)	<0.001
HbA1c, %	5.90 (5.50–6.50)	6.30 (5.75–7.15)	6.45 (5.88–7.20)	0.003
eGFR, mL/min/1.73 m^2^	99.90 ± 25.51	91.18 ± 27.54	80.25 ± 26.05	<0.001
WBC, 10^9^/L	6.31 (5.16–7.57)	6.32 (5.25–7.72)	6.40 (5.51–7.79)	0.518
RBC, 10^12^/L	4.35 (4.07–4.63)	4.28 (3.96–4.62)	4.10 (3.76–4.43)	<0.001
Hb, g/L	133.24 ± 15.23	131.76 ± 15.38	126.44 ± 17.28	<0.001
Platelet, 10^9^/L	239.86 ± 55.78	233.49 ± 62.19	231.18 ± 57.75	0.206
Neutrophil, 10^9^/L	3.52 (2.65–4.43)	3.54 (2.71–4.71)	3.73 (2.87–4.92)	0.030
Lymphocyte, 10^9^/L	2.07 (1.63–2.54)	2.01 (1.56–2.47)	1.94 (1.47–2.38)	0.021
PNR	68.48 (54.04–88.25)	65.87 (47.66–85.25)	59.19 (46.25–75.61)	<0.001
NLR	1.62 (1.20–2.22)	1.70 (1.27–2.51)	1.95 (1.44–2.79)	<0.001
PLR	113.61 (90.25–145.04)	115.10 (88.88–147.77)	123.61 (90.71–158.26)	0.188
PWR	36.55 (31.31–46.03)	36.06 (28.53–44.09)	33.55 (28.04–43.40)	0.007
PT	11.27 ± 0.73	11.33 ± 0.86	11.32 ± 0.82	0.632
PTT	31.43 ± 3.47	30.91 ± 3.98	30.45 ± 3.51	0.062
ESR, mm/h	15.89 ± 14.71	17.69 ± 15.87	21.04 ± 17.63	0.025
CRP, mg/L	2.63 ± 6.61	4.43 ± 14.16	3.81 ± 8.47	0.252

Data are expressed as mean ± SD or median (IQR), number (%).

CKD, chronic kidney disease; SAH, subarachnoid hemorrhage; HDL, high-density lipoprotein; LDL, low-density lipoprotein; HbA1c, hemoglobin A1c; eGFR, estimated glomerular filtration rate; WBC, white blood cell; RBC, red blood cell; Hb, hemoglobin; PNR, platelet-to-neutrophil ratio; NLR, neutrophil-to-lymphocyte ratio; PLR, platelet-to-lymphocyte ratio; PWR, platelet-to-white blood cell ratio; PT, prothrombin time; PTT, partial thromboplastin time; ESR, erythrocyte sedimentation rate; CRP, C-reactive protein.

* Cardiovascular disease includes coronary heart disease, heart failure, or peripheral arterial disease.

## Discussion

The main purpose of our study was to investigate whether the blood cell ratios were independently associated with the risk of IA rupture. The principal finding of our study is that PNR and PWR were significantly associated with IA rupture risk. Our findings suggest that some blood cell ratios may be independent biological markers of ruptured IA. To the best of our knowledge, the current study is the first to investigate the inter-relationship between the blood cell ratios including NLR, PLR, PNR, and PWR and rupture risk of IA.

The inflammatory response has recently been revealed as a major factor in the pathogenesis of IA. The inflammatory process is initiated by hemodynamic injury, leading to matrix metalloproteinase-mediated degradation of the extracellular matrix and apoptosis of vascular smooth muscle cells, which are the main matrix-synthesizing cells within the media of the arterial wall (22, 23). The roles of macrophages, oxidative stress, and reactive oxygen species can also be added here as contributing factors, as they are known to affect IA formation and growth (22, 23). At the same time, neutrophils, lymphocytes, various cytokines, and inflammatory mediators are involved in the phenotypic regulation of vascular smooth muscle cells (23–25). It was previously reported that neutrophil RNA was higher in patients with IA than in controls without IA, and there is also evidence that neutrophil recruitment hastens the development of abdominal aortic aneurysm (23–25). In experimental animal models, neutrophils were found to play an important role in abdominal aortic aneurysm formation and progression using a neutrophil depletion model (26). These lines of evidence demonstrate an association between neutrophils and IA. Lymphocytes are the other major inflammatory cells found in IA. T lymphocytes are the predominant cells in the aneurysm wall responsible for the production of inflammatory cytokines such as TNF-α, IFN-γ, and IL-6 (27, 28). Human aneurysm specimen studies have found T lymphocytes in the aneurysm, and T lymphocyte infiltration into the aneurysm wall has been observed in elastase-induced animal aneurysm models (27, 29). In addition, platelets have been reported to be closely associated with the mortality risk of thrombotic and hemorrhagic diseases (30). These factors and processes work together to gradually weaken the arterial wall, leading to dilation, aneurysm formation, and eventual IA rupture (22). Peripheral immunity is closely related with aneurysm rupture, and there is extensive evidence that the inflammatory cascade plays an important role in rupture. Immune cells in the blood have always been closely associated with leukocyte, lymphocyte, macrophage infiltration, and increased inflammatory mediators, which are frequently found in the walls of ruptured IA (9, 27, 31–33). Experimental mouse model study has also shown that IL-1β leads to activation of circulating leukocytes, clearly demonstrating activation of the peripheral immune system in response to IA rupture (34). Furthermore, altered expression of immune system and inflammation-related genes are frequently observed in humans and experimental models of IA, and play an essential role in the formation and rupture of IA (31).

It has been reported that higher NLR can predict lower Glasgow Coma Scale score at admission, and higher NLR values are observed in the acute phase after IA rupture (35, 36). High C-reactive protein and NLR have also been reported to be associated with mortality in patients with Stanford type A acute aortic dissection (37). In a study evaluating NLR before and after intervention, it differed significantly between patients with ruptured IA and patients with unruptured IA, and elevated NLR levels in patients with ruptured IA were associated with poor postoperative functional outcomes (38). Moreover, higher NLR at admission was reported to predict the occurrence of SAH rebleeding and poor outcome (39). Recently, it was also reported that NLR and PLR as novel inflammatory biomarkers are independent predictors of delayed cerebral ischemia development and functional outcome after acute SAH (25). Another study found that higher PNR, as well as lower PLR and NLR, was strongly associated with full functional recovery (40). In this study, the associations of PNR and PWR with rupture risk were demonstrated to be stronger than those of commonly used biomarkers such as NLR and PLR. PNR and PWR were previously reported to be biomarkers of acute ischemic stroke (41, 42). It has also been reported that PNR and PWR are associated with the degree of thrombosis and inflammation because activated platelets release chemicals related to leukocyte recruitment and interact with leukocytes and neutrophils, which exacerbate inflammation and thrombosis (43). However, these markers have not yet been comprehensively studied in IA patients. Building on these previous studies, the impact of peripheral blood cell ratios in patients with IA has been more clearly confirmed in our study.

The reason why rupture risk has a high correlation with PNR and PWR in this study is probably due to the platelet-neutrophil interaction included in PNR and PWR. Leukocytes represent the immune response while platelets primarily mediate hemostasis and thrombosis. Inflammatory immune response is usually related to platelet adhesion and the activation of the coagulation cascade (44). Because platelets interact with leukocytes at the site of vascular injury, hemostasis, thrombosis and vascular inflammation are closely linked and occur sequentially (45). These evidences may explain why the risk of IA rupture was negatively correlated with PNR and PWR.

The second major finding of our study is that higher levels of NLR and ESR and lower levels of PNR and PWR were associated with a higher PHASES score of unruptured IA subjects. Increased serum NLR was significantly associated with the size of unruptured IA and was also reported to be associated with an increased risk of poor outcome at 3–6 months (46). However, the relationship between the PHASES score, which is roughly classified according to rupture risk, and blood cell ratios has not been clearly established. The PHASES score, a scoring system for ethnity, HTN, age, aneurysm size, history of SAH of other aneurysms, and aneurysm location, has been used to predict a patient's 5-year rupture risk based on the aneurysm and patient factors described previously (20, 21). Although the PHASES scoring system is useful in assessing the risk of aneurysm rupture, most aneurysms are small and are left untreated because the risk of rupture is low and the risk of complications from prophylactic treatment generally does not outweigh the risk of this small rupture. However, many small aneurysms still rupture. The identification of small aneurysms with a high risk of rupture is important for improving their management. Therefore, to improve risk assessment and patient selection for the treatment of unruptured IA, it is necessary to identify and study the factors involved in aneurysm rupture. Considering this background, our study on blood cell ratios and rupture risk in unruptured IA should be valuable. Future prospective studies on the relationship between blood cell ratios and small aneurysm rupture risk should provide a stronger foundation supporting our findings.

The reason for using blood cell ratios is that simple blood counts are generally not sensitive enough to be used as severity-related or prognostic indicators. Analysis of certain composite parameters has been proposed to detect more subtle changes in peripheral blood associated with rupture risk. The combination of platelets, neutrophils, and leukocytes may improve the sensitivity and specificity of IA rupture risk, compensating for the low sensitivity and specificity of using one blood cell marker alone.

Several limitations of the present study need to be considered. First, this was a single-center study conducted on a single ethnic group. Because all study participants were Korean, the results cannot be generalized to other ethnicities. The results may differ across ethnicities because the prevalence of unruptured and ruptured IA is higher in Asians than in Western populations. Therefore, a large-scale multi-center external validation of the current study is necessary. Second, this was a retrospective study, so there is the possibility of selection or confounding bias. Third, we did not measure peripheral blood cell ratios or conduct brain angiography serially, which prevented us from drawing conclusions about the causal relationship between peripheral blood cell ratios and IA. Fourth, although the PHASES scoring system is widely used, it cannot definitively classify rupture risk. Finally, in this study, the shape and morphological properties of the IA were not considered. It is necessary to better measure IA rupture risk including these factors, and to verify its relationship with blood cell ratios in a prospective study.

## Conclusions

In this study, peripheral blood cell ratios were shown to have critical clinical value as new biomarkers for predicting the risk of IA rupture. In addition, our findings suggest a correlation between the peripheral blood cell ratios and the severity of IA rupture risk. If further studies clarify the relationship between blood cell ratios and IA, they could become valuable biomarkers for this condition. Thus, additional large-scale prospective studies are warranted to explore the potential mechanisms underlying these associations.

## Data availability statement

The raw data supporting the conclusions of this article will be made available by the authors, without undue reservation.

## Ethics statement

Ethical approval letter was obtained from the Institutional Human Research Ethical Committee of the CHA Bundang Medical Center (IRB approval no. CHAMC 2022-04-012). Written informed consent for participation was not required for this study in accordance with the national legislation and the institutional requirements.

## Author contributions

HK: conception, design, drafting of manuscript, acquisition of data, and final approval of manuscript. KL: conception, design, acquisition of data, analysis and interpretation of data, critical revision of manuscript, and final approval of manuscript. K-YL, S-HO, S-WC, OK, S-JN, JH, and S-HK: design, acquisition of data, revision of manuscript, and final approval of manuscript. TK: design, acquisition of data, interpretation of data, revision of manuscript, and final approval of manuscript. All authors contributed to the article and approved the submitted version.
